# Safety and Efficacy of a New Synthetic Material Based on Monetite, Silica Gel, PS-Wallastonite, and a Hydroxyapatite Calcium Deficient: A Randomized Comparative Clinic Trial

**DOI:** 10.3390/medicina56020046

**Published:** 2020-01-21

**Authors:** Javier Flores Fraile, Nansi López-Valverde, Arcadio García de Castro Andews, Juan Antonio Santos Marino, Juan M. Ramírez, Rafael Gómez de Diego, Javier Montero, Antonio López-Valverde, Leticia Alejandra Blanco Antona

**Affiliations:** 1 Department of Surgery, University of Salamanca, Instituto de Investigación Biomédica de Salamanca (IBSAL), 37007 Salamanca, Spain; j.flores@usal.es (J.F.F.); juan_santos_marino@hotmail.com (J.A.S.M.); javimont@usal.es (J.M.); anlopezvalverde@gmail.com (A.L.-V.); lesablantona@gmail.com (L.A.B.A.); 2 Science to Business Foundation, 28760 Madrid, Spain; agc@fs2b.com; 3 Department of Morphological Sciences, University of Cordoba, 14071 Cordoba, Spain; jmramirez@uco.es; 4 Department of Oral Medicine, Rey Juan Carlos University, 28922 Madrid, Spain; rgd6467@movistar.es

**Keywords:** bone substitute, post-extraction socket, alveolar preservation, controlled clinical trial

## Abstract

*Background and Objectives*: Maxillary bone defects related to post-extraction alveolar ridge resorption are usual. These defects may lead to failure in further surgical implant phases given the lack of bone volume to perform the dental implant. The objective of this clinical assay was to evaluate the safety and efficacy of an experimental synthetic bone substitute in the preservation of post-extraction maxillary alveoli. *Materials and Methods*: 33 voluntary patients who had at least one maxillary premolar tooth that was a candidate for exodontia (*n* = 39) and subsequent implant rehabilitation participated. The regenerated alveoli were monitored by means of periodic clinical examinations (days 9 ± 1, 21 ± 4, 42 ± 6, and 84 ± 6), measuring the height and width of the alveolar crest (days 0 and 180 ± 5), measurement of radiodensity using tomographic techniques (days 0–5 and 175 ± 5), and histological examination of biopsies collected at 180 ± 5 days. *Results*: No significant differences were observed during the entire follow-up period between the two groups with respect to the safety variables studied. A variation in width of −0.9 ± 1.3 mm and −0.6 ± 1.5 mm, and a variation in height of −0.1 ± 0.9 mm and −0.3 ± 0.7 mm was observed for experimental material Sil-Oss^®^ and Bio-Oss^®^, respectively. The radiodensity of the alveoli regenerated with the experimental material was significantly lower than that corresponding to Bio-Oss^®^. However, the histological study showed greater osteoid matrix and replacement of the material with newformed bone in the implanted beds with the experimental material. *Conclusions:* Both materials can be used safely and proved equally effective in maintaining alveolar flange dimensions, they are also histologically biocompatible, bioactive and osteoconductive. The experimental material showed the advantage of being resorbable and replaced with newformed bone, in addition to promoting bone regeneration.

## 1. Introduction

Maxillary bone defects caused by post-extraction alveolar resorption are among the most common causes of bone loss. This is a physiological phenomenon that occurs after dental extraction, whereby the alveolar bone crest becomes reduced in height and original width in amounts that can vary according to location and individual [1,2]. These defects can hinder the surgical phase of implant treatment due to insufficient bone volume for the proper placement of dental implants [3,4,5].

Over the past 20 years, there has been an increasing interest in a concept called “alveolar ridge preservation”, which is defined as “any procedure undertaken at the time of or after an extraction that is designed to minimize external resorption of the ridge and maximize bone formation within the socket [6]”.

In modern dentistry, it is considered that, unless certain measures are taken, this partial atrophy of the alveolar bone may compromise subsequent rehabilitation [5]. Such measures include the filling of the post extraction alveoli with autologous bone or bone substitutes, combined or not with the immediate placement of an intraosseous implant [6,7,8,9,10,11,12]. Several authors consider that autologous bone is the ideal bone substitute, although they also acknowledge the disadvantages of its limited availability and the need for additional surgery, with all its potential complications [12,13].

As an alternative, a wide variety of bone substitutes of natural or synthetic origin have been investigated, among them, those derived from equine, bovine or porcine bone, both deproteinized and demineralized, synthetic calcium phosphate ceramics, and bioglasses [13,14,15,16,17,18]. However, none of the currently available commercial bone substitutes have all the desirable characteristics, namely osteoconductivity, osteoinductivity, and biodegradability. Therefore, the search for the ideal substitute continues [18]. In this regard, a new biomaterial of synthetic origin has been produced whose biological behavior is superior to that of other existing fillers. This experimental study material is a synthetic resorbable granulate composed of monetite (Ca (1 − x) ZnxHPO_4_ 0.01 ≤ x ≤ 0.06), calcium-deficient hydroxyapatite (Ca10 − x (HPO_4_) x (PO_4_) 6 − x (OH) 2 − x) and an amorphous phase consisting of silica gel ((H_2_SiO_3_) n) and calcium phosphates [19]. It has a specific surface area of ≈50 m^2^/g and an open intragranular porosity of 60% vol. corresponding to interconnected pores with a diameter in the range of 0.01–50 μm. Its open interconnected intergranular porosity is 35%, with a pore diameter in the range of 50–300 μm. In the case of the granulate, particle size is between 0.25 and 1.00 mm. When implanted, ionic species of Ca, Si, P, and Zn that play a fundamental role in osteogenic processes are gradually released. The granules of the experimental material are progressively replaced by new bone formed both outside and inside the granules [19].

The aim of this study was to assess the safety and efficacy of a new bone substitute based on monetite, silica gel, ps-wollastonite, and a calcium deficient hydroxyapatite implanted in post-extraction alveolar sockets for the prevention of alveolar ridge resorption.

## 2. Materials and Methods

### 2.1. Experimental Design

A double blind randomized, comparative clinical trial was carried out at the Dental Clinic of the Faculty of Medicine of the University of Salamanca. The design and conduct of the study were in compliance with the ethical principles of the Declaration of Helsinki and Good Clinical Practice. The trial protocol was previously approved by the Clinical Research Ethics Committee of the Salamanca (Internal number SIL-OSS AZ001-CEIC 13/1002, date 25 March 2013) Health Area and by the Spanish Agency for Medicines and Health Products. All the recruited patients received information about the characteristics of the treatments they could receive and expressed their voluntary consent in writing.

A total of 33 patients, 13 women and 20 men, participated in the study, all of them in the 29 to 67 age range and randomly distributed into the experimental and control groups. Treatment for each patient was assigned by a randomization list automatically generated prior to the start of the study in which the treatment material is determined. In the case of bilateral treatments, treatment was assigned to one side or the other, according to a supplementary randomization list. The patient randomization was performed in order to avoid sex bias, thus the final numbers of men and women in each group were not significantly different. The patient age range was 25 to 50 years old, in this way we also limited the effect of estrogen depletion due to menopause. With the results obtained, a table was prepared, in which the code assigned to each patient was registered, and the assigned regenerative product. The number of required patients was previously estimated. The calculation was made for a bidirectional trial, considering a significance level (α) of 0.05 and a study power of 80%. A patient loss of 15% was considered, expecting a very low rate of subjects withdrawn from the study, due to the aesthetic, economic, and functional importance of implant and prosthesis placement, as well as the low incidence of episodes or expected negative effects. The calculation indicated that 30 patients were needed; 15 for the Bio-Oss group and 15 for Sil-Oss.

The experimental group was treated with the synthetic bone substitute, and the control group with Bio-Oss^®^ (Geistlich Pharma, Baden-Baden Germany) [20], which consists of bovine-derived hydroxyapatite. Both the experimental material and Bio-Oss^®^ were used in the form of 0.25–1.00 mm granules.

The main inclusion criterion was the presence of at least one maxillary premolar that was a candidate for exodontics and subsequent dental implant surgery. The exclusion criteria were: impossibility to attend follow-up visits; pregnancy and breast-feeding; mental disability that could affect understanding of the information about the study and of pre- and postoperative care instructions; HIV or hepatitis carrier state, or any disease, treatment or recent surgery that could interfere with the study; anticoagulant therapy or treatment with experimental drugs over the last month; smoking more than five cigarettes a day; dental-bacterial plaque index above 30%; severe bruxism; loss of bone border during extraction; menopause; bisphosphonate treatment; and expression of the willingness to leave the study after recruitment.

The final sample consisted of 39 maxillary premolars, of which 20 received the experimental treatment and 19 were treated with the control material (Bio-Oss^®^).

### 2.2. Surgical Procedure

In the area of the tooth to be extracted, an intracrevicular incision was made, minimally extended to the adjacent teeth, and a mucoperiosteal flap was raised 3–4 mm from the buccal and lingual bone crest. No vertical incisions were made, the purpose being to preserve as much of the interproximal papillae as possible. The extraction of the affected tooth was atraumatic, using a periosteal elevator and trying to preserve the surrounding bone walls.

**Figure 1:**
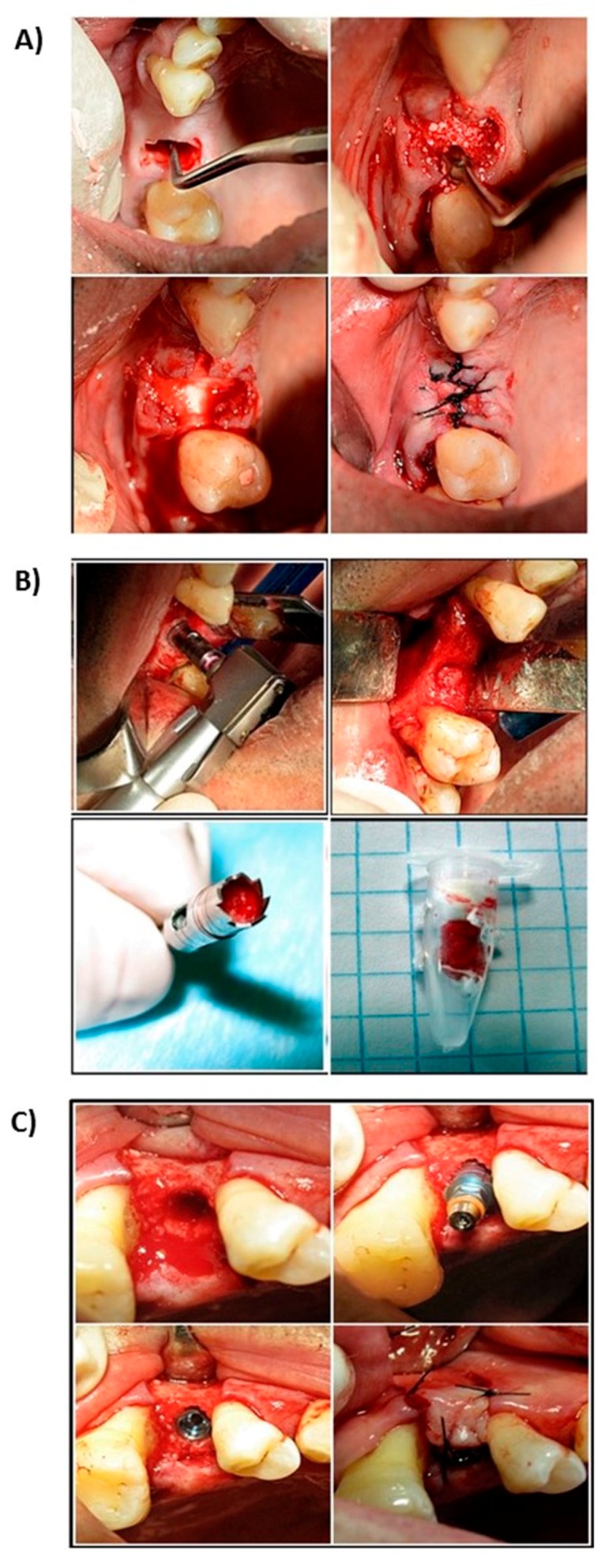
Clinical sequence surgical procedure. (**A**) Alveolus curettage after exodontist. (**B**) Stuffed with regenerative material. (**C**) Suture and closure of the socket.

Careful debridement of the alveolar cavity was performed and filled with the experimental or control material, as required, previously moistened with physiological serum, and slightly compacted, taking care not to overfill the cavity beyond the level of the bone table. The area was covered with a resorbable membrane (Osteobiol^®^ Evolution, Tecnoss, Italy) and, after replacing the flap, trying to face the edges to the maximum, it was sutured using mattress stitch (Figure 1A). At 180 ± 5 days after extraction, the area was examined again and subjected to osteoctomy using a 10 mm-long trephine milling cutter with an inner diameter of 3.5–3.7 mm (Meisinger, Neuss, Germany) to take a biopsy of the treated alveolar socket and prepare the implant bed (Figure 1B). After inserting the intraosseous implant, the flap was replaced and the tissue of the central part of the socket, previously filled with the experimental or control material, was sutured (Figure 1C).

### 2.3. Postsurgical Follow-Up

All patients received preventive antibiotic therapy (amoxicillin/clavulanic, 500/125 mg, three times a day by mouth) during the first eight days after surgery. Postoperative pain was treated with paracetamol (1000 mg) every 8 h until its remission. Patients were instructed to avoid brushing the affected area and to rinse with 0.12% chlorhexidine twice a day during the first two weeks of the postoperative period. Likewise, the use of any removable temporary prosthesis was suspended during the first three weeks of the postoperative period, prior assessment and adjustment to relieve any possible pressure on the implanted area. Patient follow-up was carried out using flange width and height measurements, together with clinical, radiographic, and histological examinations, as planned (Table 1).

**Figure 2:**
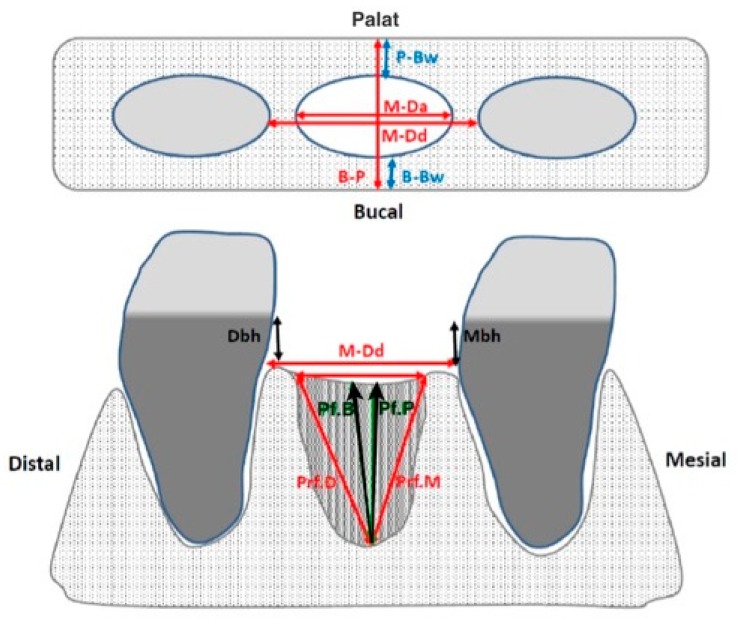
Measurements of the remaining alveolar rim performed with a millimeter probe. B-P: palatal–lingual crest width; P-Bw: palatal bone table width; B-Bw: buccal bone board width; M-Da: alveolus width; M-Dd: width of space between teeth (if both are present); Mbh: height crest cement of the adjacent mesial tooth; Ddh: height crest cement of the adjacent distal tooth; Pf.B: depth to the oral edge; Pf.P: depth to the palatal edge; Pf.M: depth to the mesial edge; Pf.D: depth to the distal edge.

**Figure 3:**
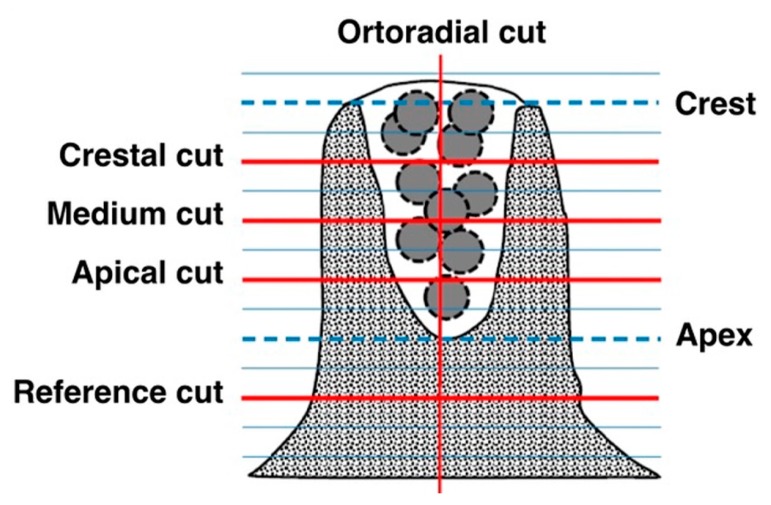
Scheme of topographic measurements used. The measurements were made on cuts parallel to the palatal vault resulting from dividing the depth of the regenerated socket between four equidistant sections: Crestal cut (Cc), Mid cut (Cm), and Apical cut (Ca). A measure is also made on the Orthoradial cut (Co). The density of the reference bone is determined from Reference cuts at 2mm from the apex of the regenerated alveolus (CRa) and in the same contralateral area (Crc).

The radiodensity of the alveoli filled with the experimental and control material was measured using computed tomography as shown in Figure 2 (PaX-Flex 3D, Vatech, Gyeonggi-do, South Korea). The cutting plane ran parallel to the palatal vault and the sweep interval was located between the alveolar ridge and the palate itself, recording and 1.5 mm thick sections with 1 mm spacing. Section selection and radiodensity measurements were performed using DicomWorks software. The measurements included total socket depth and the average of the 3 values was calculated. The radiodensity of the adjacent bone was measured in a section placed 2 mm above the alveolar apex (Figure 3). The measurements were made at 0–5 days and at 175 ± 5 days.

The trephine burs with biopsies taken at day 180 ± 5 were immersed in 4% buffered formalin for at least seven days, after which the tissue sample was extracted, trying to preserve its integrity to the maximum. The samples were washed with water and subsequently dehydrated by immersion in ethanol-water solutions with increasing ethanol concentrations. The dehydrated samples were embedded in methyl methacrylate and the sections (5 μm) were stained with Goldner trichrome.

All the clinical evaluations, namely flange width and height measurements, radiodensity measurements and histological analysis, were performed blindly by previously trained and calibrated evaluators.

### 2.4. Statistical Protocol

Data processing and statistical treatment were blinded. The statistical analysis consisted of a Chi-squared test (χ^2^) and a *t*-test performed using IBM SPSS Statistics Version 20 software (SPSS). The verified Excel sheet generated by the electronic CRD was used as input to the analysis program. The significance threshold was set at *p* < 0.05.

## 3. Results

The only adverse clinical effects reported for both treatments, as illustrated in Table 2 and Table 3, respectively, were inflammation and pain, referred during the first follow-up examination (day 9 ± 1). More than 85% of the patients reported pain and more than 70% exhibited inflammation at such point. Both symptoms can be attributed to surgical trauma, since in subsequent follow-ups they had completely remitted. The incidence of inflammation and pain was apparently higher in the control group, although no statistically significant differences were found (χ^2^, *p* > 0.05) between the experimental and control groups (Bio-Oss^®^).

During the 180 ± 5-day period between the initial and final alveolar flange dimensions measurements, both treatments yielded a small decrease in both width and height, as shown in Table 4. Flange width variation was −0.6 ± 1.4 mm in the experimental group and −0.9 ± 1.3 mm in the control group. In the case of height, the variation detected was −0.3 ± 0.7 mm for the experimental group and −0.1 ± 0.9 mm for the control group. The values obtained for experimental and control treatments were similar (no significant difference was detected, Student’s *t*-test, *p* > 0.05).

**Figure 4:**
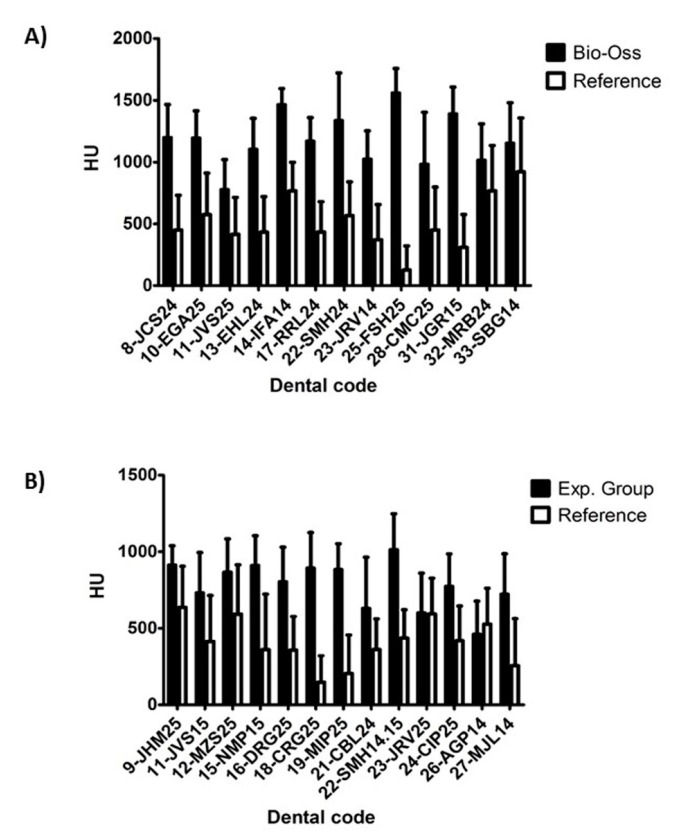
Density difference (HU) of alveoli treated with Bio-Oss^®^ (**A**), and with experimental material (**B**). The graphs represent the mean bone density in Housfield (HU) of the regenerated sites regenerated half alveoli = (Cc + Cm + Ca + Co)/4 and their corresponding reference measures: means of reference = (Cra + CRc)/2.

**Figure 5:**
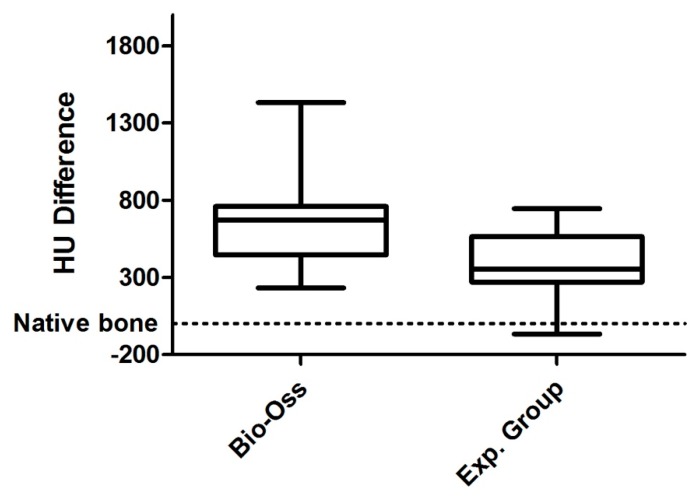
Difference in HU between Bio-Oss and Experimental group. The Whisker diagram indicating the median (inside the boxes) ± the first and third quartiles (box limits) and the error bars represented the minimum and maximum value. The values represent the difference in HU with respect to the proper bone taken as a reference (zero value) represented by the dashed line.

The degree of radiodensity at the implant sites was higher (*p* = 0.013) than on the adjacent normal bone at the two time points analyzed (Figure 4). On the other hand, radiodensity was apparently lower in the experimental group than in the control group, increasing slightly in both during the follow-up period, although such increase was not statistically significant (Figure 5).

**Figure 6:**
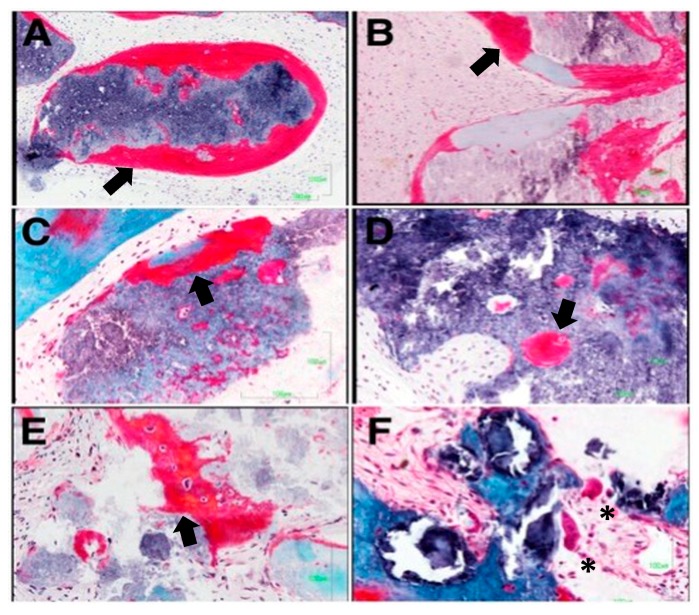
The granules of the experimental material are surrounded by bone trabeculars deficient in mineralization which corroborates their osteoforming potential (**A**,**B**). It presented greater osteinductive power, with the formation of osteoblastic ossification (arrows) fronts at the inter and intragranular levels (**C**–**E**). Additionally, in some of the samples, groups of osteoclasts are observed on the surface, evidencing the dual resorption presented by this material asterisks denote the presence of osteoclast (**F**).

**Figure 7:**
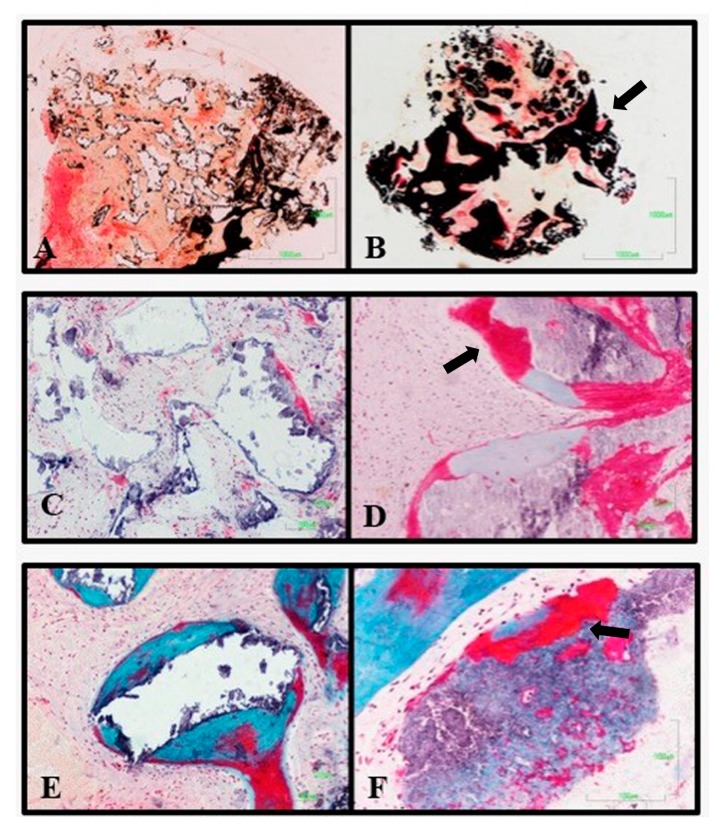
Difference in histological analysis between the Bio-Oss^®^ control material (**A**,**C**,**E**), and the experimental material (**B**,**D**,**F**). The experimental material has a higher degree of mineralization, thicker osteoid fronts and ossification centers internally of the material (arrows).

Figure 6 and Figure 7 show the most significant histological findings regarding the alveoli treated with the experimental material and with Bio-Oss^®^, respectively. Histological analysis revealed an invasion of connective tissue in the coronal area of the biopsies, both in the experimental group and in the control group. No remnants of the barrier membrane were observed in any case, suggesting that the invasion of connective tissue could be associated with its rapid resorption. In the alveoli subjected to the experimental treatment, remains of the material’s granules surrounded by abundant active osteoid matrix were detected. This was even found inside the granules surrounded by connective tissue. Accordingly, it may be assumed that the osteogenesis process would be induced by the material itself (Figure 6A,B). Most of the granules of the experimental material become completely osteointegrated by a process of partial resorption and subsequent replacement by new bone (Figure 6D). The experimental material is characterized by high intragranular porosity. The presence of pores inside the granules of the material allow cell colonization and the formation of ossification centers (Figure 6C,D). In addition to osteogenic activity (Figure 6E), osteoclastic activity can also be observed, carrying out the desired bone remodeling process (Figure 6F).

On the other hand, the histology of the alveoli that received the control treatment showed little or no variation in size and morphology of the granules of the material, showing low resorption. Greater osteoid activity was observed in the experimental group than in the control group, and this osteoid activity may be associated with the connective tissue surrounding the granules, rather than with the surface of the material. In no case was osteoid matrix observed inside the remaining granules. As in the experimental group, the granules are osteointegrated in the newly formed bone (Figure 7).

## 4. Discussion

The use of bone substitutes to produce filling for fresh post-extraction alveoli has been widely studied, accepting this therapeutic option as ideal to minimize dimensional changes in the alveolar flange [15,16]. Among the materials used as intra-alveolar bone fillers, autografts, xenografts, or other types of synthetic fillers have been used [17]. To date, all these bone fillings lack osteogenic activity, acting only as osteoconductors but maintaining the volume of the defect created after the exodontia [21].

The purpose of this study was to evaluate the safety and efficacy of the experimental biomaterial developed compared to Bio-Oss^®^ (Geistlich) in post-extraction alveolar bone regeneration. Of note, thanks to our study the European Union marking of the product was granted, and it became commercialized.

The granules of the experimental biomaterial release Ca, P, Si, and Zn ions that stimulate osteogenic activity in the implant bed, while being gradually resorbed and replaced by newformed bone tissue [22]. Preclinical trials of the experimental material in critical size defects in the proximal tibial epiphysis, middle femoral epicondyle and greater tubercle of sheep humerus demonstrated intense osteogenic activity and bone regeneration after 16 weeks [22], which is when the regeneration with new bone of 80% of the defect volume was observed [22].

On the other hand, the control treatment used in the study (Bio-Oss^®^) is a crystalline hydroxyapatite granulate obtained from deproteinized bovine bone [23]. Preclinical and clinical evidence confirm that Bio-Oss^®^ is osteoconductive, non-absorbable and remains unchanged at the implantation site, even after 11 years of implantation [23,24,25,26].

According to the results obtained in the clinical examinations conducted throughout the follow-up period, both treatments were highly safe and did not induce any chronic or severe adverse effects. The statistical analysis of the clinical variables studied (χ^2^, *p* < 0.01) yielded no significant differences between the two bone substitutes.

The reduction of buccolingual and apical-coronal dimensions of the alveolar crest after dental extraction is a consequence of the bone resorption explained by Wolff’s law, which predicts the adaptation of bone mass and structure to the intensity and frequency of the mechanical loads it is subjected to [27]. Bone resorption is greater during the first year, particularly during the first three months, and up to four times higher in the jaw than in the maxilla. The resorption of the buccal table is more marked than that of the palatal or lingual table [14]. It has been reported that, within a period of three months to one year, a height loss of 1.67 mm takes place, while the loss of width is 3.87 mm [28].

According to the results of this study, our experimental material was as effective as Bio-Oss^®^ in terms of maintaining alveolar dimensions; no statistically significant differences were found (Student’s *t*-test *p* < 0.05), between the two materials regarding alveolar ridge variations in width (experimental material −0.9 ± 1.3 mm and Bio-Oss^®^ 0.6 ± 1), 5 mm] and height (experimental material 0.1 ± 0.9 mm and Bio-Oss^®^ 0.3 ± 0.7 mm) after 180 ± 5 days (6 months). The resorption of both materials, was lower than expected in the absence of treatment [28]. The results indicate that both materials were effective in preserving the alveolar flange.

The radiodensity of the areas implanted with the experimental material was significantly lower than that of those treated with Bio-Oss^®^, both at the beginning and at the end of the follow-up. This result corresponds to the values of the X-ray linear attenuation coefficient, μ, calculated for both materials based on their chemical composition and the attenuation coefficients of their components, μi [29]. The values of μ, calculated for both materials, for X-rays with 70 keV energy, were 1136 cm^−1^ and 1511 cm^−1^, respectively, which explains the lower radiopacity observed throughout the study in the areas treated with the experimental material as compared to those treated with Bio-Oss^®^.

The biopsies taken from the implanted sites during the placement of the intraosseous implant (day 180 ± 5; 6 months) allowed a qualitative histological study, which showed bone regeneration in the alveoli treated with either material. In all cases, the amount of newly formed reticular bone was greater in the apical region, while, in the coronal region, a greater presence of loose connective tissue was observed in the remodeling process. These results correspond to the mechanism of bone regeneration and remodeling of post-extraction alveolar sockets, according to Trombelli et al. [30].

On the other hand, in terms of resorption and osteogenesis, both treatments yielded qualitatively different results. In the alveoli treated with the experimental material, there were clear signs of its resorption, specifically, the granules presented a decrease in size and an increase in porosity, always associated with the replacement of the material with new bone. Against this, there was no decrease in the size of the Bio-Oss^®^ granules. In addition, the amount of active osteoid matrix was higher in the implanted sites where the experimental study material had been used. It manifested itself not only on the surface of the granules, but also inside them, unlike in Bio-Oss^®^ granules, where only a small osteoid matrix associated with its surface was observed.

According to the above, Bio-Oss^®^ granules showed a purely osteoconductive behavior, characterized by the deposition of new bone, only on the external surface of the granules of the material, which remain unchanged and become osteointegrated into the newly formed bone matrix.

On the other hand, in the presence of the experimental material, bone neoformation is associated with both the external and the internal surface (interconnected porosity) of the granules, which were remodeled and replaced by new bone, so it could be said that, in addition to being osteoconductive, it stimulates osteogenesis mechanisms. 180 ± 5 days after the extraction and alveolar sockets treatment, both materials allowed the insertion of an intraosseous implant in the regenerated bone. Although it will be the subject of future studies, it can be stated that implanted patient evolution was favorable in both treatment groups after more than one year since the extraction and regeneration of the alveolar socket.

## 5. Conclusions

Both the tried and the control materials (Bio-Oss^®^) behaved similarly with regard to the safety variables studied: fever, pain, inflammation, suppuration and exudation of the material. No adverse effects were reported, which shows that both can be used safely. Both were equally effective in maintaining alveolar flange dimensions. However, and although our study contributed to obtain the approval for Sil-Oss^©^ commercialization, we believe that long-term studies might be designed to reassure its safety and efficacy. The radiographic density of the alveoli implanted with the experimental material was significantly lower than that of the alveoli implanted with Bio-Oss^®^, both at the beginning and at the end of the follow-up period, due to the lower intrinsic radiopacity values of the experimental material. Both the experimental material and Bio-Oss^®^ proved histologically biocompatible, bioactive and osteoconductive. The study material induced more osteoid matrix and was largely replaced by newformed bone. The experimental material is just as effective as Bio-Oss^®^ in maintaining the alveolar flange, with the advantage of being resorbable and replaced with newformed bone, in addition to promoting bone regeneration.

## Figures and Tables

**Table 1:** Action protocol carried out for each phase of the study.

Visit	Days
Recruitment visit	Up to 20 days prior to treatment
Surgical treatment visit. First surgery phase	Day 0
Post-operative TAC-visit	Day 0–5
Suture withdrawal visit	Day 8–10
3 Week follow-up visit	Day 21 ± 4
6 week follow-up visit	42 ± 6
12 week Follow-up visit	84 ± 6
TAC visit pre-implant 25 weeks	175 ± 5
Visit Biopsy and placement of implant at 26 weeks. Second stage	180

**Table 2:** Statistical analysis for Inflammation in both groups.

Infl.	Bio-Oss	Exp. Group	Total	Eb	Es	O-Eb	O-Es	O-Es2/Es	O-Eb2/Eb	χ^2^
0	1	6	7	3.410	3.590	−2.410	2.410	1.703	1.618	4.839 *p* > 0.5
1	17	14	31	15.103	15.897	1.897	−1.897	0.238	0.226	
2	1	0	1	0.487	0.513	0.513	−0.513	0.540	0.513	
Total	19	20	39					2.482	2.358	

Infl.: Inflammation.

**Table 3:** Statistical analysis for Pain in both groups.

Pain	Bio-Oss	Exp. Group	Total	Eb	Es	O-Eb	O-Es	O-Es2/Es	O-Eb2/Eb	χ^2^
0	1	3	4	1.949	2.051	−0.949	0.949	0.462	0.439	1.975 *p* > 0.5
1	17	17	34	16.564	17.435	0.436	−0.436	0.011	0.011	
2	1	0	1	0.487	0.513	0.513	−0.513	0.540	0.513	
Total	19	20	39					1.013	0.963	

**Table 4:** Height and width crest variation.

	Wide Variation		Height Variation	
	Bio-Oss	Experimental	Bio-Oss	Experimental
Mean ± s.d.	−0.88 ± 2.14	−0.57 ± 2.53	−0.125 ± 1.65	−0.312 ± 1.31
*t*-test; (*p*)	−0.345; 0.732		0.356; 0.724	
